# Clocks in the Wild: Entrainment to Natural Light

**DOI:** 10.3389/fphys.2020.00272

**Published:** 2020-04-02

**Authors:** Christoph Schmal, Hanspeter Herzel, Jihwan Myung

**Affiliations:** ^1^Department of Biology, Faculty of Life Sciences, Institute for Theoretical Biology, Humboldt Universität zu Berlin, Berlin, Germany; ^2^Department Basic Sciences, Institute for Theoretical Biology, Charité – Universitätsmedizin Berlin, Berlin, Germany; ^3^Graduate Institute of Mind, Brain, and Consciousness, Taipei Medical University, Taipei, Taiwan; ^4^Brain and Consciousness Research Centre, Taipei Medical University-Shuang Ho Hospital, Ministry of Health and Welfare, New Taipei City, Taiwan; ^5^Graduate Institute of Medical Sciences, Taipei Medical University, Taipei, Taiwan; ^6^Computational Neuroscience Unit, Okinawa Institute of Science and Technology Graduate University, Okinawa, Japan

**Keywords:** circadian clock, entrainment, photoperiodism, seasonality, chronotype, Arnold onion

## Abstract

Entrainment denotes a process of coordinating the internal circadian clock to external rhythmic time-cues (Zeitgeber), mainly light. It is facilitated by stronger Zeitgeber signals and smaller period differences between the internal clock and the external Zeitgeber. The phase of entrainment ψ is a result of this process on the side of the circadian clock. On Earth, the period of the day-night cycle is fixed to 24 h, while the periods of circadian clocks distribute widely due to natural variation within and between species. The strength and duration of light depend locally on season and geographic latitude. Therefore, entrainment characteristics of a circadian clock vary under a local light environment and distribute along geoecological settings. Using conceptual models of circadian clocks, we investigate how local conditions of natural light shape global patterning of entrainment through seasons. This clock-side entrainment paradigm enables us to predict systematic changes in the global distribution of chronotypes.

## 1. Introduction

Many organisms on Earth are subject to a precise 24 h light-dark rhythm. To predict the cycle, most organisms including humans maintain their own timekeeping system, the circadian clock running at a close-to-24 h period without external time cues. An organism's internal clock synchronizes to the daily cycles of the external environment through light as a time-giving cue, called Zeitgeber. The proper coordination of intrinsic rhythms with environmental cycles upon entrainment contributes to a better evolutionary fitness and health (Ouyang et al., 1998; Dodd et al., 2005; Chen et al., 2010). Elucidating entrainment under realistic conditions of intrinsic (clock properties) and extrinsic (light) factors can provide chronobiological insights into geoecological dynamics. Natural light conditions on Earth vary by latitude and seasons. Given the individual variability of circadian periods, either due to natural variation within the same species or due to differences between species, it is likely that entrainment produces a global patterning of circadian phases that are rich in diversity. We address this question by combining mathematical core clock models with geophysical data on seasonal and latitudinal light intensities.

### 1.1. Mathematical Clock Models

At the organismal level, circadian clocks exhibit properties of endogenous limit cycle oscillators, i.e., their rhythms have a well-defined period as well as amplitude under constant conditions and are robust against perturbations. In the mammalian core clock, tissue-level coupling leads to a precise pacemaker (Herzog et al., 2004), even though cellular rhythms of its constituent single neurons are relatively imprecise and partially arrhythmic (Hirata et al., 2019). Perturbations of these organismic rhythms, by light pulses, pharmacological treatments or jet-lag usually decay at relatively fast time scales of a few days (Spoelstra et al., 2004; Kiessling et al., 2010; Ono et al., 2017). Under particular circumstances, external perturbations under constant conditions can even stop the clock (singularity behavior) (Winfree, 1970; Engelmann et al., 1978).

Conceptual models make predictions on the dynamical behavior of circadian clocks based on heuristic principles (e.g., the notion of limit cycle behavior) without considering the detailed molecular machinery that leads to circadian rhythm generation at the cellular level. Such conceptual modeling has a long tradition in chronobiology and helped to understand the response of clock dynamics to external perturbations (Engelmann et al., 1978; Peterson, 1980), entrainment schedules (Wever, 1964), or synchronization processes (Kronauer et al., 1982; Liu et al., 1997), to name a few.

In contrast to this, contextual or detailed biochemical models aim to decipher the molecular regulatory machinery (i.e., the interaction of genes, mRNAs, proteins, post-translational modifications, etc.) that leads to rhythm generation within a biological context, specific to different tissues (Pett et al., 2018) or different organisms such as cyanobacteria, plants, fungi, flies, and mammals (Goldbeter, 1995; Hong et al., 2006; Locke et al., 2006; Kim and Forger, 2012; Woller et al., 2016).

### 1.2. Photoperiodic Entrainment of Circadian Clocks

Unidirectional synchronization of an endogenously oscillating system like the circadian clock to an external periodic forcing signal (Zeitgeber) is termed entrainment. Upon entrainment, a circadian clock with an internal free-running period τ is forced onto the external Zeitgeber period *T* (period locking). By this means, a stable phase of entrainment ψ emerges as the internal rhythm aligns with the external rhythm (phase locking). The range of entrainment denotes the set of periods that are able to entrain to a Zeitgbeber at a given strength. It usually gets wider (or narrower) with increasing (or decreasing) Zeitgeber strength. The entrainment region within the parameter plane spanned by the period detuning τ − *T* and Zeitgeber strength *Z* adopts a tongue like geometry, called *Arnold tongue* (Arnold, 1987).

Entrainment ranges, phases of entrainment ψ and entrained amplitudes vary systematically not only with respect to Zeitgeber but also with intrinsic clock properties (Aschoff, 1960). Entrainment protocols have therefore been extensively used to compare circadian clocks in different tissues (Abraham et al., 2010), in different genetic background (clock mutants) within the same species (Rémi et al., 2010; Erzberger et al., 2013), or to compare circadian systems of different species (Aschoff and Pohl, 1978).

**Figure 1:**
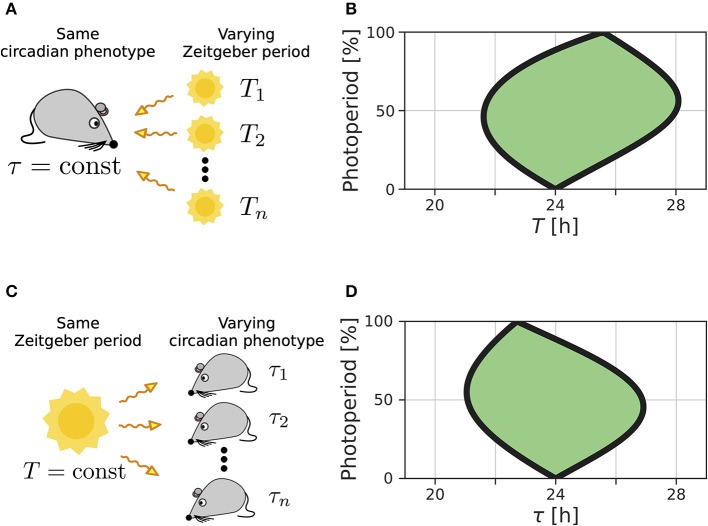
Entrainment ranges vary with photoperiod. **(A)** A certain circadian phenotype with free running period τ is entrained to Zeitgeber cycles of varying *T*. **(B)** Entrainment region (green) of a self-sustained oscillator under entrainment with varying photoperiods ϰ and Zeitgeber periods *T*. The circadian free-running period under constant darkness (ϰ = 0) has been set to τ = 24 h. **(C)** Different circadian phenotypes with varying free-running period τ, due to variation in a population of organisms or genetic background (e.g., clock mutants), entrained by a Zeitgeber signal with a fixed period *T*. **(D)** For a given Zeitgeber period *T* = 24 h, the entrainment region (green) can be determined for varying free-running periods τ and different photoperiods ϰ. Simulations underlying panels **(B)** and **(D)** have been done by simulating Equations (4) and (5), applying a square-wave Zeitgeber signal of peak-trough amplitude 0.1 to the *x*-variable and by using oscillator parameters *A* = 1 and λ = 0.5 h^−1^ as described in Schmal et al. (2015).

We recently extended the concept of Arnold tongues to account for seasonality (Schmal et al., 2015). If we consider a circadian system entrained by Zeitgbeber signals of varying period *T* and photoperiod ϰ (Figure 1A), the region of entrainment adopts an onion shaped geometry (Figure 1B), called *Arnold onion*. Here, the photoperiod ϰ has been defined as the fraction (in percent) of illumination time (daylength) over the period *T* of the Zeitgeber cycle. While the entrainment range is largest during equinoctial Zeitgeber cycles (ϰ = 50%) it tapers toward the free-running periods under constant darkness (ϰ = 0%) and constant light (ϰ = 100%) for increasingly extreme photoperiods. Since the free running periods are generally not the same under constant light and constant darkness as predicted by Aschoff's rule (Aschoff, 1960), the Arnold onion appears to be tilted (compare Figure 1B).

A similar but conceptually different situation occurs if we consider entrainment of organisms with various circadian free-running periods τ to Zeitgeber signals of fixed period *T* but varying ϰ (Figure 1C). Diverse circadian periods exist due to natural variation within a population of the same species or traceable clock gene mutations (Konopka and Benzer, 1971). The entrainment range in the free-running period τ and photoperiod ϰ parameter plane adopts an onion shaped geometry similar to the above described case while being tilted in the opposite direction, compare Figures 1B,D.

Here, we extend our analysis to the Earth's natural light conditions from photoperiodic entrainment as originally described in Schmal et al. (2015), where we investigated entrainment to square wave Zeitgeber signals, corresponding to typical laboratory conditions. Using a conceptual model of the circadian clock in combination with a simple celestial mechanics-based approximation of local light signals, we illustrate circadian entrainment under ecologically relevant conditions. This reveals a global map of how organisms entrain under different seasons and at different latitudes. It also proposes a possibility that the distribution of chronotypes, a quantitative proxy of the phase of entrainment, can change throughout the year and at different positions on the Earth.

## 2. Results

### 2.1. The Poincaré Oscillator: A Conceptual Model for Limit Cycle Behavior

**Figure 2:**
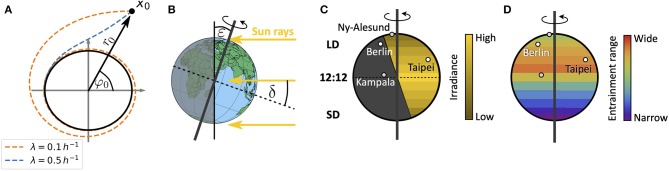
Schematics of entrainment range prediction through geophysical considerations. **(A)** Phase plane representation of the conceptual clock model, the Poincaré oscillator. While the black circle denotes the limit cycle of steady state amplitude *A*, dashed lines show transient dynamics for different values of the radial relaxation rate λ, starting from an initial condition *r*_0_ and φ_0_ or *x*_0_ = (*r*_0_cos(φ_0_), *r*_0_sin(φ_0_)). Larger values of λ lead to shorter transient dynamics after perturbations. **(B)** Illustration of the celestial constellation between the Earth and Sun during summer in the northern hemisphere. Here, ε denotes the obliquity of the ecliptic while δ is the declination of Sun. **(C)** Global distribution of daylengths on a summer day in the northern hemisphere, with geographical locations of representative cities used in this study: Kampala (equator), Taipei (sub-tropics), Berlin (temperate), and Ny-Alesund (above arctic circle). Note that the irradiance is lower in the northern extreme despite the longer daylength. LD, long daylength; 12:12, equinox; SD, short daylength. **(D)** Combining a conceptual oscillator model with a celestial mechanics based approximation of the light intensity, we compute entrainment ranges and phases of entrainment ψ under different seasons and latitudes. By this we can study impact of season and latitude on the distribution of chronotypes.

Circadian clocks share several properties of limit cycle oscillators at the tissue or organismal level. They cause rhythms of gene expression and physiological processes that are stable in period and amplitude, robust against (small) external perturbations. In order not to restrict ourself to a specific biological context (i.e., a particular organism or cell type) we omit detailed biochemical reactions and exploit a generic amplitude-phase model, whose dynamics is determined by

(1) $$\begin{array}{rcl} \frac{\text{d}r}{\text{d}t} & = & \lambda r\left(A-r\right) \end{array}$$

(2) $$\begin{array}{rcl} \frac{\text{d}\varphi }{\text{d}t} & = & \frac{2\pi }{\tau } \end{array}$$

in radial coordinates. Equations (1, 2) describe a Poincaré oscillator (Glass and Mackey, 1988), which exhibits limit cycle oscillations of explicitly defined steady state amplitude *A* and free-running period τ. Here, *r*(*t*) describes the dynamics of the radial component while φ(*t*) describes the angular component. Radial-relaxation rate λ determines the time-scale of transient dynamics, i.e., the time a Poincaré oscillator needs to return to its steady-state orbit after an amplitude perturbation (e.g., through light pulses) (Figure 2A). While parameter dependencies of oscillator properties such as the amplitude or free-running period are usually intertwined in complex molecular models, the Poincaré oscillator (1, 2) conveniently treats these features as independent parameters. Thus, the impact of internal clock parameters on entrainment and synchronization properties can be studied in a straightforward manner as demonstrated in several studies (Abraham et al., 2010; Granada et al., 2013; Gu et al., 2014; Tokuda et al., 2015; Myung et al., 2018; Schmal et al., 2019).

In the following we will represent the circadian clock of an organism by a Poincaré oscillator (1, 2) and study its entrainment under natural light environments, using a simple celestial mechanics derivation.

### 2.2. Approximation of the Perceived Light Intensity on Earth

For a sessile observer at a given position on Earth, the Sun obeys an apparent movement across the celestial sphere. This movement leads to changes in light intensity, predictable from celestial mechanics. While the rotation of the Earth around its own axis leads to daily changes of light intensity, the tilt ε (obliquity of the ecliptic) of its rotation axis with respect to the plane that is spanned by the Earth's orbit around the Sun (the ecliptic) leads to seasonal variations (Figure 2B).

A simple celestial mechanics based model for the light intensity *I*, perceived by an organism at latitude ϕ and on a given day *N* of the solar year, assumes that the declination δ of the Sun (see Figure 2B) varies sinusoidally

(3) $$\begin{array}{r} \delta \approx \varepsilon \sin\left(\frac{2\pi \left(N-80\right)}{365.2422}\right) \end{array}$$

across the year, thus neglecting the elliptical motion of the Earth around the Sun (Khavrus and Shelevytsky, 2012). Here, 365.2422 denotes the mean number of days in a year, i.e., the mean orbital period of the Earth. The ecliptic ε is subject to fluctuations at timescales that are extremely long (Milanković cycles) compared to any organism's life expectancy such that we consider it constant. It currently takes a value of about ε = 23.44°. In addition to celestial constellations between the Earth and the Sun, scattering and absorption of light within the atmosphere further affects the solar radiation that reaches an observer at the Earth's surface. The extent of absorption, reflection and scattering depends on the path length within the Earth's atmosphere, called the optical air mass (AM), which in turn depends on latitude as well as season (Khavrus and Shelevytsky, 2012).

The orientation of the light-perceiving surface (e.g., the retina or plant leaves) with respect to the direction of the Sun affects the solar irradiance as well. Combining all of these considerations, we can give a closed form expression for the solar irradiance perceived at any position on Earth in dependence on latitude ϕ, time of year *N*, time of day and the orientation toward the Sun, as given by Equation (11) in section 4.

**Figure 3:**
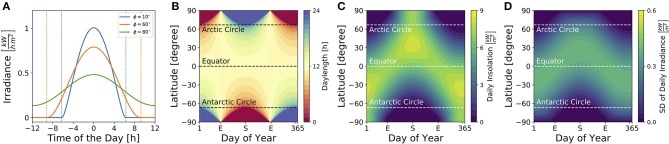
Impact of season and latitude on daylength and light intensity. **(A)** Solar irradiance on the Earth's surface for three different latitudes [ϕ = 10° (blue), ϕ = 60° (orange), ϕ = 80° (green)] on day *N* = 172 which corresponds to the 21st of June, i.e., summer solstice in the northern hemisphere. Here, time point zero corresponds to solar noon, i.e., when the Sun reaches the highest point at sky. Dashed lines correspond to sunrise and sunset as given by condition (8). **(B)** Daylength for different latitudes and times of year are depicted as given by Equation (9) in section 4. The daylength corresponds to the distance between sunrise and sunset as exemplified in **(A)** for three different latitudes. **(C)** Daily insolation for different latitudes and times of year. The daily insolation is defined as the daily sum over the solar irradiance as depicted in **(A)** for three exemplary latitudes. Please note that the solar irradiance in **(A)** and solar insolation in **(C)** have been calculated for sun rays reaching a horizontal plane. The direction of the surface (e.g., of plant leaves, the retina, etc.) has an impact on the solar irradiance perceived. **(D)** Standard deviation of the solar irradiance, calculated over a complete day (24 h), for different latitudes and times of year.

Figure 3 illustrates dependencies of the solar irradiance (A), daylength (B), total daily insolation (C) and standard deviation of the daily solar irradiance (D) on geographical latitudes and seasons for sun rays reaching a horizontally oriented surface. While the daylength (see Equation 9) is constantly 12 h throughout the year at the equator as well as at vernal and autumnal equinoxes for all latitudes ϕ, it adopts the highest variation between different latitudes at summer and winter solstices (Figure 3B). The total daily insolation (i.e., the summed irradiance per day) is determined jointly by daylength and irradiance. At summer solstice, for example, the daily insolation adopts similar values at the equator and the arctic pole since a high irradiance is perceived over a shorter daylength near the equator, while a low irradiance is perceived over a long 24 h photoperiod at the arctic pole (Figure 3C).

Critical for circadian entrainment is the variation of a Zeitgeber signal, which can be quantified by the standard deviation of the solar irradiance over one day (Figure 3D). Its highest values are reached during the equinoxes at the equator and during summer solstice at temperate latitudes, while the lowest variation is found during polar night. These local geographical Zeitgeber variations hint at global variations of the entrainment range.

### 2.3. Entrainment Ranges Vary Systematically With Season and Latitude

Circadian clock properties vary among different species and even among individuals of the same species as a result of natural (genetic and epigenetic) variation. This eventually translates into variations of entrainment properties such as the phase of entrainment ψ, even within the same species that receive the same entrainment cues. Such emergence of different “temporal phenotypes” can be represented as a spread of entrainment phases ψ in a population of individuals of same species. One such representation is commonly known as chronotypes (Pfeffer et al., 2015). In the following we investigate how organisms with different internal clock properties as described by the conceptual oscillator model (1, 2) entrain to natural light signals at different seasons and geographical locations.

**Figure 4:**
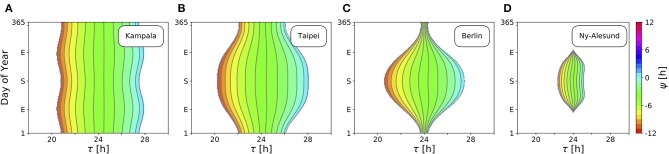
Entrainment ranges narrow down at high latitudes. Depicted are the entrainment regions and color-coded phases of entrainment ψ for different times around the year and varying intrinsic free-running periods τ at four exemplary latitudes ϕ, namely, ϕ = 0.3° **(A)**, ϕ = 25.03° **(B)**, ϕ = 52.51° **(C)**, ϕ = 78.92° **(D)**, corresponding to the latitudes of Kampala, Taipei, Berlin, and Ny-Alesund, respectively. Regions without color-coded phases correspond to parameter combinations of *N* and τ where the circadian clock is unable to entrain to the external Zeitgeber cycle. Other intrinsic oscillator properties have been set to *A* = 6 and λ = 0.1 h^−1^.

Figure 4 depicts ranges of internal free-running periods τ, for which entrainment upon natural light signals given by Equation (11) can be observed, at different times of the year *N* and at four different representative latitudes ϕ, namely in Kampala close to the equator (A), Taipei in the sub-tropics (B), Berlin at a temperate latitude (C), and Ny-Alesund (D) above the arctic circle. Here, we only consider the effect of free-running period τ. Intrinsic clock properties other than τ are held constant at *A* = 6 and λ = 0.1 h^−1^ throughout all simulations. Phases of entrainment ψ have been color-coded within the regions of entrainment. Solar noon, the highest position of the Sun at the celestial sphere, is used as a reference point such that ψ = 0 h is adopted if the acrophase of the circadian clock oscillations (given by *x*(*t*)) coincides with solar noon.

Close to the equator, there is little per-day change of daylength, insolation and variation (standard deviation; SD) throughout the year in comparison with higher latitudes (Figures 3B,D). Thus, entrainment ranges remain relatively constant throughout the year (Figure 4A). Within these small variations, the largest range of entrainment occurs twice a year at vernal and autumnal equinoxes while it becomes the smallest around summer and winter solstices. This is in line with the profiles of solar insolation and SD of daily irradiance, peaking twice per year close to the equator (Figures 3C,D).

At higher latitudes, this half-year period in insolation and SD of daily irradiance is absent, leading to entrainment ranges that are maximal during summer solstice and minimal at winter solstice (Figures 4B,C). The seasonal effect is stronger as the latitude ϕ is higher. At high latitudes, organisms with a circadian clock whose free running period τ substantially deviates from 24 h might be able to well entrain only during summer but could fail to entrain during winter season, as described for European hamsters (*Cricetus cricetus*) kept under natural lighting conditions (Monecke and Wollnik, 2005).

At latitudes above the arctic circles, the Sun never rises during polar night and never sets during midnight sun (Figure 3B). Since our approximation of light intensity ignores diffraction effects by the atmosphere, organisms are unable to entrain during the polar night (Figure 4D). Between the vernal and autumnal equinoxes, entrainment is generally possible even during a 24 h midsummer day, that is because the solar altitude above the horizon still exhibits diurnal variations. In line with these results, reindeers (*Rangifer tarandus*) in Spitzbergen do not show diurnal rhythmicity during winter but some residual diurnal activity throughout the summer season (Arnold et al., 2018). Other species such as Svalbard ptarmigans (*Lagopus mutus hyperboreus*) show behavioral arrhyhtmicity during polar night and midnight sun (Williams et al., 2015).

**Figure 5:**
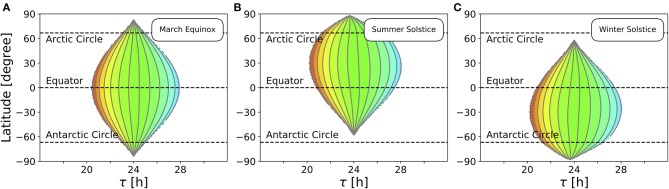
Entrainment depends on latitude and time of year. Entrainment regions and color-coded phases of entrainment ψ for different latitudes and internal free-running periods τ at three different times of the year, namely March equinox **(A)**, summer solstice **(B)** and winter solstice **(C)**. Other internal clock parameters have been set to *A* = 6 and λ = 0.1 h^−1^. Color coding of ψ is the same as in Figure 4.

So far, we studied the range of entrainment throughout the year at four different exemplary latitudes (Figure 4). In Figure 5, we investigate the effect of varying latitudes at three different times of the year. Since the daylength is approximately 12 h at all latitudes (Figure 3B) and the dependency of the Zeitgeber intensity (SD of irradiance) on latitude is symmetric for the northern and southern hemispheres, the entrainment region during vernal equinox (and analogously at autumnal equinox) is perfectly symmetric (Figure 5A). The largest entrainment range is adopted at the equator (ϕ = 0°) and tapers toward the polar regions. At summer solstice in the northern hemisphere, an asymmetric entrainment region can be observed (Figure 5B). Due to polar night no entrainment is possible above the antarctic circle during this season, but it is possible above the arctic circle. The largest entrainment range is adopted around ϕ ≈ 30° as the SD of daily irradiance becomes maximal (compare Figure 3D). Since summer solstice in the northern hemisphere corresponds to winter solstice on the southern hemisphere and *vice versa*, the entrainment region at winter solstice in the northern hemisphere is a mirror image of the summer solstice entrainment region (Figure 5B) with respect to the equator (Figure 5C).

### 2.4. Tracking of Dusk and Dawn Depends on Free-Running Period τ

From an evolutionary point of view, two aspects of circadian entrainment need to be considered. On the one hand, it has to be assured that the circadian program entrains to the environmental Zeitgeber signals at all, which is related to the range of entrainment. Of equal importance, on the other hand, is the existence of a proper phase of entrainment ψ, ensuring that physiological processes are executed at most beneficial times around the solar day.

**Figure 6:**
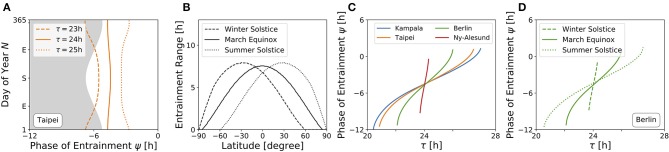
High sensitivity of phases during winter and at high latitudes. **(A)** Phase of entrainment ψ at the latitude of Taipei (ϕ = 25.03°) vs. time of the year (*N*) for three different internal free-running periods τ = 23 h (dashed line), τ = 24 h (bold line), and τ = 25 h (dotted line), respectively. The gray shaded area depicts the duration of darkness. **(B)** Entrainment ranges with respect to variations in τ as determined by the difference between the upper and lower limits of entrainment in Figures 5A–C for different latitudes, respectively. **(C)** Dependency of the phase of entrainment ψ on internal free running period τ on March equinox for the latitudes of Kampala (blue), Taipei (orange), Berlin (green), and Ny-Alesund (red). **(D)** Dependency of the phase of entrainment ψ on internal free running period τ at winter solstice (dashed line), March equinox (bold line), and summer solstice (dotted line) at the latitude of Berlin (ϕ = 52.51°). All simulations have been performed for light perceived by a horizontal plane. Phases of ψ = ±12 h and ψ = 0 h correspond to midnight and solar noon, respectively.

An internal oscillator's activity onset phase can be locked to different phases around the Zeitgeber reference. This is because ψ depends on Zeitgeber properties as well as internal oscillator properties such as the free-running period and amplitude. Figure 6A depicts the evolution of ψ throughout the year for three different free running periods τ (23, 24, and 25 h) at the latitude of Taipei (ϕ = 25.03°), corresponding to vertical cross-sections through the entrainment regions as depicted in Figure 4B. While circadian oscillators with an internal period of 24 h (bold line, Figure 6A) appear to be phase locked in parallel to solar noon throughout the year, ψ for clocks with an intrinsic period τ longer than 24 h (dotted line, Figure 6A) occurs earlier during summer days compared to winter and has its earliest value at summer solstice. The opposite is true for circadian clocks with τ < 24 h, where ψ occurs later during summer in comparison to winter days (dashed line, Figure 6A). These results quantitatively confirm a similar qualitative description by Pittendrigh (Pittendrigh, 1988). The biological importance can be interpreted within the framework of Aschoff's rule which states that day-active animals typically have a period longer than 24 h while night-active animals have a period shorter than 24 h (Aschoff, 1958). Thus, ψ of day active animals (τ > 24 h) follows the earlier sunrise as photoperiods increase in spring and summer, while night-active animals (τ < 24 h) rather follow a later dusk (onset of night) as photoperiods increase (Pittendrigh, 1988) (compare Figure 6A).

### 2.5. Phases of Entrainment ψ Vary Systematically With Season and Latitude

The range of entrainment depends on Zeitgeber strength and photoperiod. It has been shown that, within the range of period detunings τ − *T* that allow for entrainment at a given fixed Zeitgeber strength and photoperiod, the phase of entrainment ψ varies by about 180° (Wever, 1964; Granada et al., 2013; Schmal et al., 2015). This translates to a higher sensitivity of ψ with respect to period detunings τ − *T* for small ranges of entrainment.

Figure 6B shows the dependency of entrainment ranges on latitude, exemplarily for March equinox as well as for northern hemisphere's summer and winter solstices. It can be seen that the entrainment range gradually decreases as the latitude gets higher. From this, we can infer that on March equinox, ψ has an increased sensitivity to τ variations as the latitude increases (Figure 6C). This corresponds to horizontal cross-sections at March equinox (*N* = 79) in Figures 4A–D or to horizontal cross-sections at the latitudes of Kampala (equator), Taipei, Berlin and Ny-Alesund (above arctic circle) in Figure 5A, respectively. Likewise, the phase of entrainment ψ has the highest sensitivity to τ variations (*T* = 24 h) at winter solstice, the lowest sensitivity at summer solstice, and an intermediate sensitivity at March equinox. This is illustrated for the latitude of Berlin (ψ = 52.51°) in Figure 6D, which corresponds to horizontal cross-sections at the respective times of the year in Figure 4C. A similarly strong dependency of the slope of ψ on the Zeitgeber strength has been described for the entrainment of lizards to temperature cycles (Hoffmann, 1969).

The sensitivity of ψ with respect to τ variations can be used to explain the behavior of chronotypes across different seasons and latitudes as investigated in the following section.

### 2.6. Season and Latitude Affect the Spread of Chronotypes

In our analysis of generic clocks, ψ is referenced to the point of midday. This referencing is arbitrary and is easily translatable to chronotypes. Human chronotype reflects the phase of entrainment, and is commonly referenced to the mid-sleep phase. Variations in the free-running period τ have been shown to translate into changes of the phase of entrainment ψ (Winfree, 2001; Bordyugov et al., 2015). Genetically modified strains of *Neurospora crassa* with relatively shorter and longer τ, respectively, exhibit early and late values of ψ under light entrainment (Rémi et al., 2010). Likewise, the free-running period of fibroblasts is positively correlated with phase ψ under temperature entrainment (Brown et al., 2008). This has also been seen in weakly coupled *Bmal1* oscillators in the suprachiasmatic nucleus explants cultures (Myung et al., 2012).

Along these lines, it has been hypothesized that small variations of free-running periods τ within a population of the same species can lead to the emergence of different chronotypes as variations in the phase of entrainment (Phillips et al., 2010; Granada et al., 2013). Chronotypes based on sleep-wake time questionnaires can be a proxy to the phase of entrainment ψ, although in higher organisms other factors such as a homeostatic sleep drive interferes with pure circadian effects. “Strong” clocks with a small entrainment range and a large sensitivity of ψ will map a given spread of free-running periods τ (described by standard deviation σ_τ_) into a relatively large spread of chronotypes (σ_ψ_) when compared to weak clocks with a large entrainment range and a small sensitivity of ψ.

**Figure 7:**
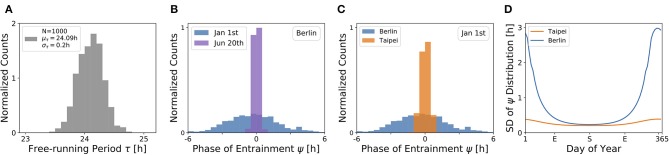
The distribution of chronotypes broadens in winter and at higher latitudes. **(A)** Distribution of free-running periods τ sampled from a normal distribution of mean μ_τ_ = 24.09 h and standard deviation σ_τ_ = 0.2 h, following what has been described for female human using a forced desynchronization protocol (Duffy et al., 2011). **(B)** Distribution of entrainment phases ψ (i.e., of chronotypes), that follows from a period distribution as depicted in **(A)** under natural light entrainment in winter (Jan 1st) and summer (Jun 20th) at the latitude of Berlin (ψ = 52.51°). Approximately 12% of the (most extreme) sampled periods τ do not properly entrain under the conditions on Jan 1st. **(C)** Distribution of entrainment phases ψ during winter (Jan 1st) in Berlin (blue) and Taipei (orange). **(D)** Systematic analysis of the standard deviation in the distribution of ψ throughout the year at the latitude in Berlin (blue) and Taipei (orange). In the computations underlying panels **(B–D)**, oscillator parameters others than τ are held constant to *A* = 6 and λ = 0.1 h^−1^.

Under a forced desynchronization protocol, it has been shown that human females adopt a distribution of internal free-running periods of mean μ_τ_ = 24.09 h and standard deviation σ_τ_ = 0.2 h (Duffy et al., 2011). Taking this data as an illustrative example, we sampled 1, 000 periods from a normal distribution with this mean and standard deviation (see Figure 7A) and computed the corresponding entrainment phases ψ at different seasons and latitudes. This task corresponds to a convolution of the period distribution with the phase of entrainment profiles as determined in Figures 4, 5, 6C,D.

The effect of season on the distribution of chronotypes in our model has been exemplarily calculated for the latitude of Berlin (Figure 7B). In summer (Jun 20th), a strong Zeitgeber signal (large SD of irradiance) leads to a wide entrainment range and a small sensitivity of ψ with respect to τ variations. In other words, the distribution of chronotypes is relatively narrow (σ_ψ_). In winter (Jan 1st), light becomes a relatively weak Zeitgeber, leading to a narrow entrainment range and a high sensitivity of ψ. This ultimately leads to a larger spread in chronotypes (σ_ψ_) in winter compared to summer.

If the same population would travel during winter season (Jan 1st) from the latitude of Berlin to latitudes substantially closer to the equator, for example Taipei, the population would find the distribution of chronotypes to become narrower (Figure 7C). That is due to the fact that the Zeitgeber is stronger toward the equator in winter (Figure 3D), eventually leading to a wider entrainment range and a smaller sensitivity of ψ with respect to τ variations (see Figure 5C in the northern hemisphere).

These theoretical observations imply that the spread in chronotypes (σ_ψ_) varies throughout the year. Examples in Figure 7D have been calculated, based on the τ distribution in Figure 7A, the temperate latitude of Berlin and the latitude of Taipei closer to the equator. It can be seen that the seasonal effect is relatively smaller at the latitude of Taipei than in Berlin. At the higher latitude, a standard deviation of σ_τ_ = 0.2 h in free running periods leads to a standard deviation of up to almost σ_ψ_ = 3 h in the distribution of ψ during winter solstice.

**Figure 8:**
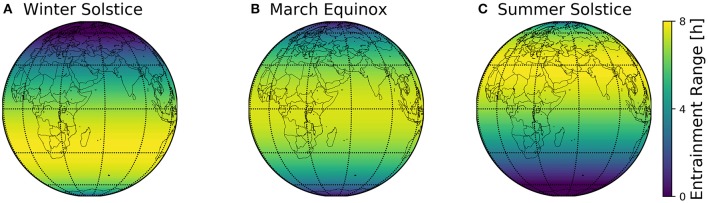
Global entrainment patterns across seasons. Entrainment ranges at different latitudes ϕ are depicted on a globe map for three different times of the year, namely winter solstice **(A)**, March equinox **(B)**, and summer solstice **(C)**. This representation follows the scheme outlined in Figure 2, where the entrainment ranges correspond to those depicted in Figure 6B.

The combined geographical distributions of entrainment ranges throughout the seasons can be mapped on the globe, using the scheme outlined in Figure 2. This visualizes which regions on the globe are easier to entrain to local day-night conditions (Figure 8).

## 3. Discussion

The major finding of the present study is the organization of circadian phases in dependence of the geographical and seasonal context and individual clock properties (personal chronotypes). This organization can be assessed by a construct we call *Arnold onion* (Schmal et al., 2015). The clock's ability to entrain to natural light-dark cycles, i.e., the entrainment range, reorganizes across the year, which can be most intuitively visualized for different latitudes on the globe (Figure 8). By combining a generic clock model with a geophysical model of local light intensities, we could examine season- and latitude-dependent entrainment properties for circadian systems with different clock properties.

The range of internal periods τ, capable of entraining to daily rhythms of the outside world, remains relatively stable close to the equator but shows strong seasonal variations at high latitudes, having a narrower entrainment range during winter (Figure 4). This theoretical prediction is consistent with experimental studies, showing stronger entrainment during summer compared to winter where entrainment might even fail at higher latitudes (Monecke and Wollnik, 2005; Arnold et al., 2018).

For a given circadian phenotype with a well-defined internal period τ and given amplitude, our model predicts systematic variations of the phase of entrainment ψ across the seasons. Similar to earlier studies (Pittendrigh and Daan, 1976b; Pittendrigh, 1988), dusk tracking is predicted for clocks shorter than a day (τ < 24 h) while dawn tracking occurs for clocks with τ > 24 h (Figure 6A). Such seasonal variations in the timing of daily activity have been described for entrainment under natural conditions in several species including birds and rodents (Aschoff and Wever, 1962; Daan and Aschoff, 1975). In some species, other entrainment cues such as social synchronization and temperature can overtake photic entrainment (Robbers et al., 2015; Fuchikawa et al., 2016).

Our theoretical examination predicts that there can be latitudinal and seasonal changes in the global distribution of entrainment phases ψ, which can be reflected in chronotype measures. Season and latitude can affect the mean (Figure 6) and standard deviation (Figure 7) of the chronotype distribution as the average internal free-running periods (μ_τ_) deviate from 24 h. In humans, distributions of the midpoint of sleep on free days (MSF), a common measure of chronotypes, are different between male and female populations and vary also with age (Foster and Roenneberg, 2008). Humans adopt their latest chronotype roughly at the age of 20, and become earlier again with increasing age (Roenneberg et al., 2004). Reliable estimates of population variance through seasonal and latitudinal variations are difficult to obtain amidst these internal heterogeneities, in addition to confounding cultural influences amongst studies including societies of different lifestyles (Pilz et al., 2018). We expect that finer details of chronotype diversity can be predicted in defined subpopulations of age and gender when more precise physiological mechanisms of chronotypes are known. It should be noted that our model has been developed to exploit general principles of circadian entrainment and has not been fine-tuned to account for human entrainment characteristics. Human chronotypes are commonly associated with the “preferred sleep-wake schedule,” and the sleep-wake cycle is not linearly related to the underlying circadian cycle. Along with the circadian regulation, human sleep patterns are simultaneously affected by homeostatic processes (Phillips et al., 2010; Skeldon et al., 2016). Previous conceptual models of human circadian entrainment more specifically elaborated on light-sensing mechanisms and mimicked human phase-response behavior (Forger et al., 1999; Jewett et al., 1999). It would be interesting to investigate in future studies how these human-specific constraints affect the season and latitude dependent responses of chronotype distributions. However, the occurrence of chronotypes is not exclusive to humans and has been described for other species as well (Pfeffer et al., 2015). Our theoretical predictions could be useful for further studies on the effect of detailed Zeitgeber characteristics upon the distribution of chronotypes or entrainment ranges within a controlled setting, using populations of well-studied model organisms at different geographical locations and seasons.

Parameters other than free-running periods τ such as the amplitude have been proposed to further affect entrainment characteristics (Lakin-Thomas et al., 1991). Throughout this study we held amplitudes (*A*) and radial relaxation rates (λ) constant and focused on the impact of τ on entrainment properties. Analogous to previous studies (Abraham et al., 2010; Bordyugov et al., 2011) we find that decreasing amplitudes and relaxation rates broaden the entrainment range with respect to τ variations throughout the year (Figures S1, S2). Therefore, decreasing amplitudes and relaxation rates will lead to a reduced spread of entrainment phases. A careful interpretation of entrainment characteristics among different circadian phenotypes (organisms, tissues, mutants, etc.) will require inclusion of a thorough analysis of all relevant internal oscillator parameters.

Properties of the circadian clock have been shown to be plastic and diverse within the same species and across an organism's domicile environment and life span. Chronic conditions of external entrainment can alter the intrinsic period of a clock (Pittendrigh, 1960; Pittendrigh and Daan, 1976a). Across several organisms, including mice and human, it has been shown that entrainment at different photoperiods (Ciarleglio et al., 2011; Myung et al., 2015) and different Zeitgeber periods *T* (Scheer et al., 2007; Azzi et al., 2014, 2017) reversibly or irreversibly changes the free-running period τ, a phenomenon termed aftereffect or imprinting, respectively. Additionally, it has been found that properties of the clock change with increasing age (Pittendrigh and Daan, 1974). Although our conceptual oscillator model does not include light- or age-dependent parameter plasticities, Arnold onions can be used to predict changes of entrainment properties upon plasticities in the circadian clock behavior by highlighting subpopulations of τ. Assuming that plasticities occur either under relatively short (after-effects) or long (aging) time scales in comparison to seasonal changes of light intensity, one can predict plasticity dependent changes of ψ in a latitude and season dependent manner by tracing relevant τ subpopulations expected from plasticities within an Arnold onion as depicted in Figures 4, 5.

Latitudinal clines of oscillator properties have been observed in various organisms. Plant leaf movements and oviposition or eclosion rhythms in Drosophila show shorter free-running periods in northern compared to southern strains (Mayer, 1966; Hut et al., 2013). The free-running period in tomato decelerates during domestication, most probably to adapt to longer summer days after its transferal from the equator to northern latitudes (Müller et al., 2015). Our conceptual clock model, extended to account for photoperiodic entrainment under natural conditions, can help predict how properties of the internal clock affect the phase of entrainment and thus can help to untangle mechanisms underlying latitudinal clines.

Several approximations have been done with respect to the calculated light intensity that entrains the conceptual clock model. We assumed that a given organism perceives light similar to a horizontal plane. Young sunflowers follow the path of the sun across the celestial sphere during the day, driven by anti-phasic patterns of stem elongation (Vandenbrink et al., 2014; Atamian et al., 2016). By this means, a larger amount of daily insolation can be perceived as compared to a horizontal leaf orientation (Shell et al., 1974; Figure S3A). The variation of the light intensity per day, on the other hand, increases with solar tracking (Figure S3B) which facilitates the entrainment of the circadian system especially during winter (Figure S3C). Active orientation toward the Sun can therefore lead to broader entrainment ranges and a narrowed distribution of chronotypes. Future studies might investigate the impact of orientation toward the light source on entrainment characteristics in more detail.

Light intensity as perceived by an organism on Earth has been calculated as the sum over the whole spectrum of sunlight throughout this study. It is known that some species harbor different wavelength of natural light differentially (Roenneberg and Foster, 1997; Fankhauser and Staiger, 2002). Changes of spectral composition throughout the course of the day have been proposed to serve as an entrainment cue (Walmsley et al., 2015). In modern societies, anthropogenic artificial light interferes with the natural light-dark profiles. Self-selected light during the night has been described to affect the sleep-wake behavior in humans (Skeldon et al., 2017; Pilz et al., 2018). In addition, changing weather and movement between differentially shaded habitats affect the amount of light that a given organism perceives. An extension of our current study could investigate how above described effects on the effective Zeitgeber strength alters our computationally estimated entrainment properties under natural conditions.

The entrainment phase depends on the detuning (τ − *T*) of the clock and Zeitgeber period, on the ratio of the Zeitgeber strength to clock amplitudes, and on relaxation rates (Wever, 1964; Granada et al., 2013; Bordyugov et al., 2015; Schmal et al., 2015). These generic dependencies theoretically allow us to predict entrainment properties as a function of season and latitude. It is therefore expected that our modeling approach will provide a framework to analyze empirical data on chronotype distributions under different geographical and ecological contexts.

## 4. Materials and Methods

### 4.1. Conceptual Amplitude-Phase Model

Using the identities *x* = *r*cos(φ) and *y* = *r*sin(φ), we can rewrite Equations (1, 2) of the *main text* as

(4) $$\begin{array}{rc} \frac{\text{d}x}{\text{d}t} & =\lambda x\left(A-r\right)-\frac{2\pi }{\tau }y \end{array}$$

(5) $$\begin{array}{rc} \frac{\text{d}y}{\text{d}t} & =\lambda y\left(A-r\right)+\frac{2\pi }{\tau }x \end{array}$$

in Cartesian coordinates. Similar to previously published studies (Granada et al., 2013; Schmal et al., 2015), we choose that light affects the circadian clock by adding the light intensity *I*(*t*) at a given time *t* to the *x* direction on the phase plane such that Equation (4) reads as $\frac{dx}{dt}=\lambda x\left(A-r\right)-\frac{2\pi }{\tau }y+I\left(t\right)$.

### 4.2. Light Intensity on a Cloud-Free Day

Considering a plane of arbitrary orientation with a polar angle β and the azimuth angle γ, the cosine of the angle θ between the normal of the arbitrarily oriented plane and the direction of the Sun is given by

(6) $$\begin{array}{rc} \cos\left(\theta \right)= & \sin\left(\delta \right)\left[\sin\left(\phi \right)\cos\left(\beta \right)-\cos\left(\phi \right)\sin\left(\beta \right)\sin\left(\gamma \right)\right]\\ \;+ & \cos\left(\delta \right)[\cos\left(\phi \right)\cos\left(\beta \right)\cos\left(\omega \right)+\sin\left(\phi \right)\sin\left(\beta \right)\\ \;\cos\left(\gamma \right)\cos\left(\omega \right)+\sin\left(\beta \right)\sin\left(\gamma \right)\sin\left(\omega \right)]. \end{array}$$

where ϕ, δ and ω are the geographical latitude, declination and hour angle, respectively (Chen, 2011). The hour angle ω defines the solar time and grows by 2π between two subsequent crossings of a meridian.

From Equation (6), one can obtain the cosine of the zenith angle *z*, i.e., [the angle between the zenith and the Sun disk], by setting β = 0, i.e.,

(7) $$\begin{array}{r} \cos\left(z\right)=\sin\left(h\right)=\cos\left(\delta \right)\cos\left(\omega \right)\cos\left(\phi \right)+\sin\left(\delta \right)\sin\left(\phi \right), \end{array}$$

with *h* being the solar altitude. At sunrise and sunset the sun just touches the horizon (*h* = 0) such that the hour angle ω_*s*_ fulfills the relationship

(8) $$\begin{array}{r} \cos\left(\omega_{s}\right)=-\tan\left(\delta \right)\tan\left(\phi \right), \end{array}$$

from which we can obtain the daylength

(9) $$\begin{array}{r} D=24h\left(1-\frac{\arccos\left(\tan\left(\delta \right)\tan\left(\phi \right)\right)}{\pi }\right) \end{array}$$

at a given latitude ϕ and declination δ of the Sun (Khavrus and Shelevytsky, 2010; Chen, 2011).

Using a common approximation for the optical air mass (AM) (Kasten and Young, 1989)

(10) $$\begin{array}{r} \text{AM}={\left[\cos\left(z\right)+0.50572{\left(96.07995-z\right)}^{-1.6364}\right]}^{-1} \end{array}$$

where z is the zenith angle in degrees, we can write the total irradiance *I* that reaches a plane horizontally-aligned to the Earth's surface as

(11) $$\begin{array}{l} I=\{\begin{array}{l} 1.353\frac{kW}{m^{2}}\times 1.1\times 0.7^{AM^{0.678}}\times \cos\left(z\right)\;\text{if }\cos\left(z\right)>0\\ 0\;\text{otherwise} \end{array} \end{array}$$

where $1.353\frac{kW}{m^{2}}$ is the average solar constant and factor 1.1 is for diffuse irradiance (Khavrus and Shelevytsky, 2012).

### 4.3. Numerics

Equations (4, 5) in combination with Equation (11) have been numerically solved using the SCIentificPYthon function odeint at a step size of Δ*t* = 0.01 h. Entrainment ranges as well as phases of entrainment have been determined as described earlier (Schmal et al., 2015). The highest position of the Sun at the celestial sphere served as a reference point of the Zeitgeber cycle such that the phase of entrainment ψ ∈ [−12*h*, 12*h*] is defined as the distance between the acrophase of *x*(*t*) oscillations and solar noon.

## Data Availability Statement

All datasets generated for this study are included in the article/Supplementary Material.

## Author Contributions

CS designed the study, performed the analysis, visualized the data and drafted the manuscript. JM designed the study, made a figure, and drafted the manuscript. All authors interpreted the data and wrote the manuscript. All authors read and approved the submitted version.

### Conflict of Interest

The authors declare that the research was conducted in the absence of any commercial or financial relationships that could be construed as a potential conflict of interest.
