# Supplementary material for: Clocks in the Wild: Entrainment to Natural Light
Source: Front Physiol. 2020 Apr 2;11:272. doi: 10.3389/fphys.2020.00272 (PMC7142224; doi:10.3389/fphys.2020.00272)
Supplement: Supplementary file 1 [file Data_Sheet_1.pdf]

frontiers

Supplementary Material

1 SUPPLEMENTARY FIGURES

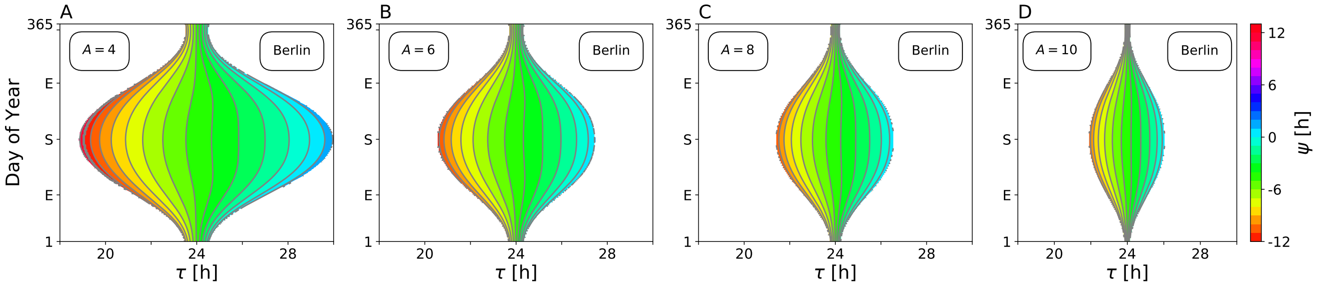

Figure S1. Entrainment region decreases with increasing clock amplitude. Entrainment regions and color-coded phases of entrainment ψ for $\phi = 52.51°$, a latitude corresponding to Berlin, for four different free running amplitudes A of the internal clock (i.e. the Poincaré oscillator), namely $A = 4, 6, 8$ and 10 in panels A-D, respectively.

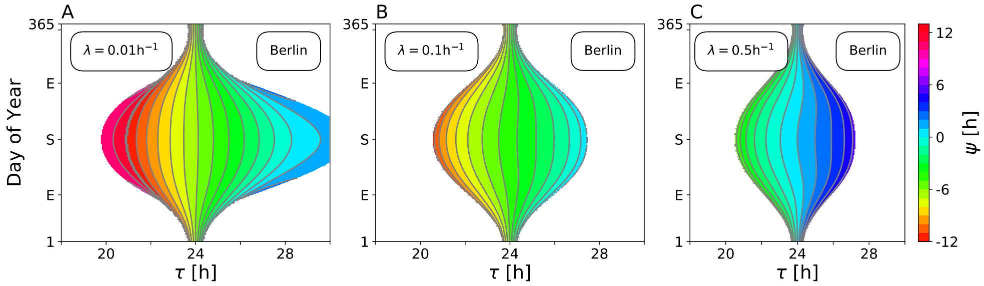

Figure S2. Entrainment region decreases with increasing radial relaxation rate. Entrainment regions and color-coded phases of entrainment ψ for $\phi = 52.51°$, a latitude corresponding to Berlin, for three different radial relaxation rates λ of the internal clock (i.e. the Poincaré oscillator), namely $\lambda = 0.01\ \text{h}^{-1}, 0.1\ \text{h}^{-1}$ and $0.5\ \text{h}^{-1}$ in panels A-C, respectively.

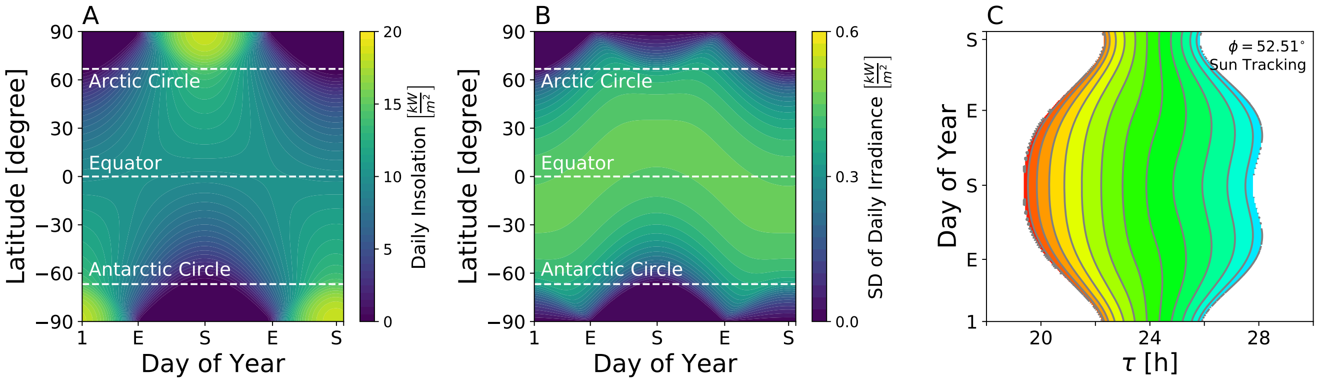

Figure S3. Sun tracking enhances the perceived daily insolation and leads to a robust entrainment at winter season. A) Daily insolation for different latitudes and times of year in case of a Sun tracking light perceiving surface. B) Standard deviation of the solar irradiance, calculated over a complete day (24h), for different latitudes and times of year in case of a Sun tracking light perceiving surface. C) Entrainment regions and color-coded phases of entrainment ψ for $\phi = 52.51°$, a latitude corresponding to Berlin, in case that the circadian clock perceives light analogously to a Sun tracking surface. Compare Figures 3 B and D as well as Figure 4 C of the *main text*.
